# Oxygen physiology and mechanisms of oxygen toxicity: a narrative review

**DOI:** 10.4103/mgr.MEDGASRES-D-25-00140

**Published:** 2026-01-23

**Authors:** Danyong Liu, Ting Li, Qinjun Chu, Jianyu Zhu, David Dewei Xia, Xia Li, Chunyan Wang, Zhengyuan Xia

**Affiliations:** 1 Department of Anesthesiology, Affiliated Hospital of Guangdong Medical University, Zhanjiang, Guangdong Province, China; 2 Department of Anesthesiology, Shenzhen Maternity and Child Healthcare Hospital, Women and Children’s Medical Center, Southern Medical University, Shenzhen, Guangdong Province, China; 3 Department of Anesthesiology and Perioperative Medicine, Zhengzhou Central Hospital Affiliated to Zhengzhou University, Zhengzhou, Henan Province, China; 4 Faculty of Kinesiology & Physical Education, University of Toronto, Toronto, Canada; 5 Department of Anesthesiology, Renmin Hospital of Wuhan University, Wuhan, Hubei Province, China; 6 Doctoral Training Platform for Research and Translation, Boshiwan, Zhongxiang, Hubei Province, China; 7 State Key Laboratory of Pharmaceutical Biotechnology, Department of Medicine, The University of Hong Kong, Hong Kong Special Administrative Region, China

**Keywords:** elderly, hyperoxia, hypoxia, mitochondria, oxidative phosphorylation, oxygen, oxygentoxicity, reactive oxygen species, sex

## Abstract

**Facts**

1. Oxygen is essential for most living organisms on the Earth, but excessive oxygen can cause oxygen toxicity.
2. For individuals with mitochondrial dysfunction, even normal oxygen concentration in the air may be relatively excessive.
3. Consensus regarding oxygen supply for critically ill patients in the intensive care unit has yet to be reached.

**Open Questions**

1. How to strike a balance between insufficient and excessive oxygen supply during oxygen inhalation?
2. Is it necessary to integrate monitoring of oxygen supply to form a closed-loop oxygen supply system with autonomous regulation for patients/individuals who need oxygen therapy?
3. How to better achieve individualized oxygen supply?

Oxygen inhaled through respiration is consumed in the mitochondria, mainly for oxidative phosphorylation to produce energy. Too little or too much oxygen can be extremely harmful to humans. Insufficient oxygen supply to tissues and organs can result in either dysfunctions or necrosis. However, when the oxygen supply is over supplied, the body is unable to consume the excessive oxygen, which puts the cells in a state of hyperoxia, leading to the production of a large number of reactive oxygen species, which can further cause oxidative damage to the cell membranes and organelles, leading to oxygen toxicity. Although the body has several oxygen-sensing mechanisms to prevent organs and cells from being exposed to hypoxia- or hyperoxia-induced oxidative stress, the relevant capacity and duration of action are relatively limited. Thus, continuous and real-time individualized monitoring and guidance is particularly important in oxygen therapy, especially in the elderly, in order to correct hypoxemia and tissue hypoxia while avoiding or reducing oxygen toxicity caused by hyperoxia. This review aims to briefly summarize the physiology of oxygen and to update the latest progress regarding the mechanism of oxygen toxicity, providing theoretical insights on oxygen therapy practice.

## Introduction

Oxygen is a chemical element abundant in the Earth’s biosphere and is critical for the evolution of life on the Earth. Oxygen is primarily involved in the energy conversion of photosynthesis and aerobic respiration, thereby maintaining homeostasis within the Earth’s biosphere, particularly in organic life. Humans obtain the required oxygen from the air by breathing and continuously deliver it to all cells in the body, where it participates in the metabolic process of the intra-mitochondrial tricarboxylic acid (TCA) cycle and ultimately produces energy.

However, different levels of partial pressure of oxygen are observed in different tissues in the body, and oxygenation of tissues relies on oxygen delivery and consumption. For example, the thymus, leukocytes^1^,^2^ and other tissues consume less oxygen, whereas the brain, which weighs only 2% of the body weight but consumes 20% of the total oxygen the body needed.^3^,^4^ When certain tissues and organs in the body are in a situation of insufficient supply or impaired utilization of oxygen, it will lead to abnormal changes in their metabolism, function and structure, and this phenomenon is called hypoxia.^5^ Hypoxia is usually defined as a state in which the whole body (generalized hypoxia) or a region of the body (localized hypoxia) is deprived of adequate supply of oxygen at the tissue level, including disturbances in oxygen utilization. This condition is usually caused by a reduction in the amount of oxygen carried by hemoglobin in the blood, or a reduction in the partial pressure of oxygen in the blood below a certain level. Oxygen deprivation can have very detrimental effects on the body, and the brain in particular is particularly sensitive to oxygen deprivation.^3^ Prolonged cerebral hypoxia may lead to brain cell dysfunction or even death, and hypoxia may also lead to cyanosis of the skin and lips, chest tightness, shortness of breath and respiratory distress.^5^

In the early 20^th^ century, supplemental oxygen was commonly used as a medical treatment. However, excess oxygen may induce a hyperoxic state in the organism, which may generate excessive reactive oxygen species (ROS) through mitochondrial metabolism, leading to deleterious oxidative stress and even cellular dysfunction or death.^6^ The present review summarized the advancements on the pathophysiological mechanisms underlying the metabolism of oxygen in the body.

## Search Strategy

We searched the research in PubMed by the key words (“oxygen” and “mitochondria”) or (“oxygen” and “exercise” or “oxygen”) and (“heart” or “oxygen”) and (“age” or “oxygen”) and (“sex” or “oxygen”) and (“critically ill patients” or “oxygen”) and “oxygen toxicity,” for publications published between 2000 and 2025. The inclusion criteria for the literature were as follows: (a) Types of studies: original research articles, including randomized controlled trials, cohort studies, case-control studies, and basic science experiments (*in vitro* and *in vivo*), systematic reviews and meta-analyses; and (b) studies published in the English language. The studies for which the full text cannot be retrieved were excluded.

## Mitochondria in Oxygen Metabolism

Approximately 98% of the body’s oxygen is consumed in the mitochondria, mainly for the TCA cycle and oxidative phosphorylation (OXPHOS), where most of the oxygen is used for OXPHOS to generate energy, and a small portion is used to generate ROS and heat.^7^ OXPHOS is an important cellular process that uses oxygen and monosaccharides, among other things, to produce adenosine triphosphate (ATP), the cell’s main source of energy.^8^

Mitochondria have two distinct features in their use of oxygen: (1) They use oxygen to rapidly form water, which leads to hypoxia in closed environments; (2) their consumption of oxygen to generate cellular energy, heat and ROS.^9^ Mitochondria are often regarded as oxygen sensors that respond differently to low and high concentrations of oxygen.^9^,^10^ Under hypoxic conditions, cells provide energy primarily through the glycolytic pathway rather than through mitochondrial metabolism which consumes oxygen molecules. At this time, the flux of the TCA cycle in the mitochondria is reduced, leading to a reduction in metabolites required for anabolic processes. Hypoxia can affect mitochondria by causing mitochondrial fusion, fission, autophagy, and further affect OXPHOS.^11^ It also affects the activity and function of the electron transport chain (ETC), including the regulation of different mitochondrial ETC complexes and the amount of nicotinamide adenine dinucleotide and flavin adenine dinucleotide recycling equivalents in the TCA cycle.^10^ Notably, the rate-limiting threshold for ETC activity is 0.3% of the intracellular oxygen level.^12^ Mitochondrial complex IV is the terminal complex in the ETC that delivers four electrons to oxygen, producing two molecules of water.^13^ Thus, ETC can function in a condition that is near to hypoxia, whereas cells can largely maintain ATP levels in short-term hypoxia. Although short periods of mild hypoxia do not severely inhibit ETC function, prolonged or short periods of severe hypoxia lasting more than a few hours can reduce ETC function. Notably, hypoxia has been shown to reduce the enzymatic maximal velocity of isolated cyclooxygenase, suggesting an intrinsic oxygen dependence of cyclooxygenase during prolonged hypoxia.^13^,^14^

**Figure 1:**
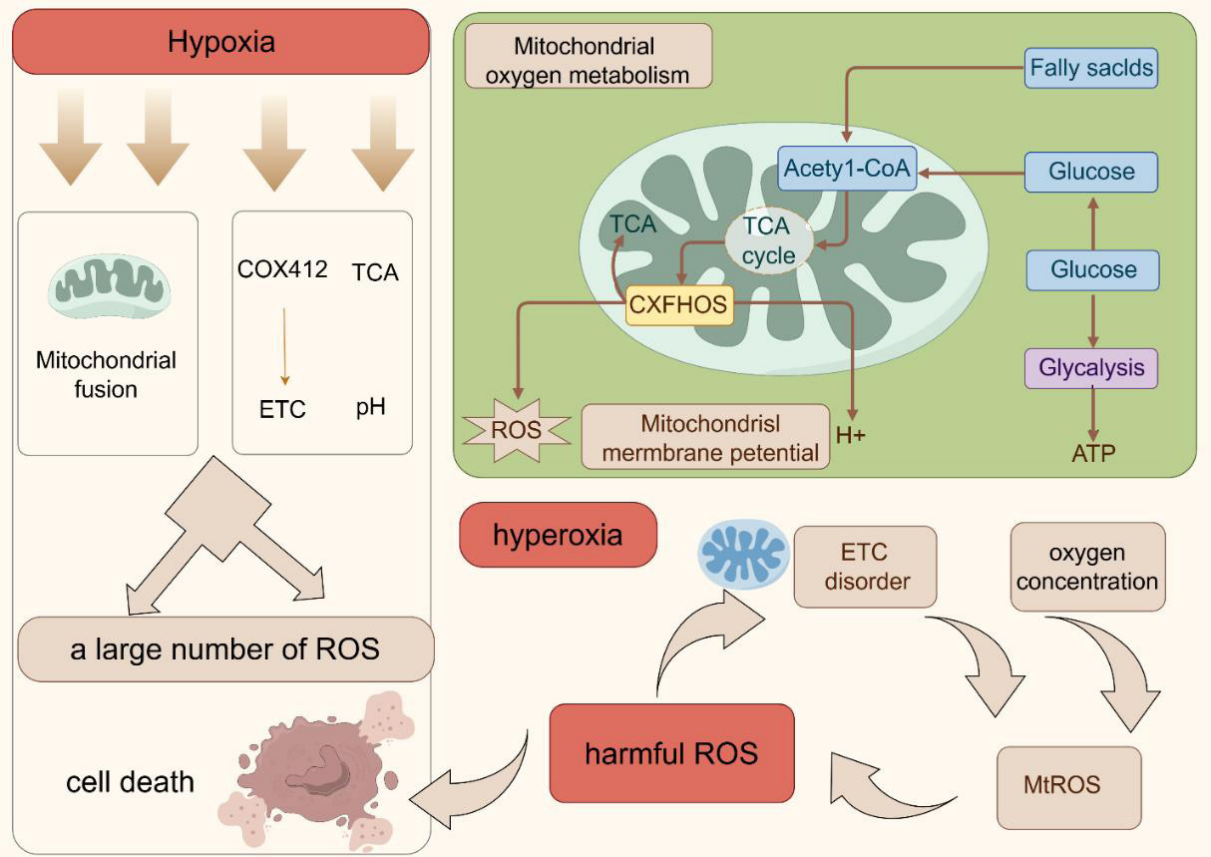
Mechanisms of cell death under either hypoxic or hyperoxic condition. Both hypoxia and hyperoxia can lead to excessive ROS production. Excess ROS can oxidize almost all biomolecules, including proteins, DNAs (including the nucleus and mitochondria), lipids and carbohydrates, leading to cellular damage. In addition, oxidative damage to mitochondrial DNA by ROS leads to defective synthesis of ETC subunits, and damaged mitochondria tend to produce more ROS, which activates mitochondria-mediated apoptosis or necrosis pathways. Created with Figdraw 2.0 (ID: RAPWO3f9cc). ATP: Adenosine triphosphate; ETC: electron transport chain; MtROS: mitochondrial reactive oxygen species; ROS: reactive oxygen species; TCA: tricarboxylic acid cycle.

In humans, exposure to hyperoxic conditions is routinely administered to patients for supplemental oxygen therapy. Under hyperoxia, proteins other than hemoglobin gain the ability to carry oxygen, which increases the ability of oxygen to diffuse from the plasma into the mitochondria of various cells.^15^ During OXPHOS, mitochondrial ROS production occurs mainly at the ETC on the inner mitochondrial membrane. However, mitochondrial ROS production is also dependent on the oxygen concentration, and the rate of mitochondrial ROS production increases linearly with the increase of intracellular oxygen concentration.^16^ Low concentrations of ROS are essential for a variety of physiological signaling pathways.^17^,^18^ However, there exists a threshold range above which ROS become harmful.^19^,^20^ Excess ROS oxidizes almost all biomolecules, including proteins, DNA (including the nucleus and mitochondria), lipids and carbohydrates, leading to cellular damage.^10^ In addition, oxidative damage to mitochondrial DNA by ROS leads to defective synthesis of ETC subunits, and damaged mitochondria tend to produce more ROS, which activates mitochondria-mediated apoptosis or necrosis pathways^21^,^22^ (**Figure 1**).

## Oxygen in Exercises

Typically, skeletal muscle OXPHOS is a key component of circulatory regulation in response to oxygen and energy demand during exercise. The increased oxygen demand of working muscles for aerobic energy production is met by increased circulatory delivery of oxygenated blood to the muscles and increased levels of muscle oxygen extracted from the blood, but an individual’s peak work capacity is limited by the cardiovascular capacity to deliver oxygen to working muscles.^23^

Healthy individuals also experience a net increase in oxygen extraction levels relative to oxygen delivery during exercise from venous blood by working muscles.^24^,^25^ This is also indicated by the exercise-related decrease in oxygen partial pressure levels, which is consistent with an increase in the rate of oxygen utilization by respiratory mitochondria relative to the rate of increase in oxygen delivery. Thus, the difference in the body’s arteriovenous oxygen partial pressure difference represents the mitochondrial capacity for OXPHOS. In healthy individuals, the arteriovenous oxygen partial pressure difference increases from 5 mL/dL at rest to 15 mL/dL at peak exercise and does not limit oxygen utilization during exercise.^26^ In contrast, circulating oxygen delivery is thought to be a major determinant of aerobic exercise, but the exact mechanism by which oxygen delivery is closely matched to oxygen utilization is unknown. It may involve the activation of neural reflexes via metabotropic receptors in working muscles that respond to metabolites reflecting the oxidative demands of the muscle. The extent to which oxygen delivery and oxygen extraction influence the increase in muscle oxygen uptake during dynamic exercise is a matter of ongoing debate. It has been proposed that local muscular factors, such as capillary beds and mitochondrial oxidative capacity, play an important role in prolonged low-intensity training of small muscle groups when there is no direct limit to cardiac output.^27^ In addition, the magnitude of the increase in maximal aerobic capacity determines exercise capacity. Under normal air conditions, the process of delivering oxygen to the muscles involves complex regulatory mechanisms, which depend on the concentration of hemoglobin and the muscle perfusion status.^28^ In vertebrates, hemoglobin in the blood is mainly responsible for transporting oxygen from the lungs to the tissues to support aerobic metabolism of cells.^29^ Given that the amount of oxygen physically dissolved in the blood is much lower than the oxygen demand of the tissues,^30^ the transport of oxygen by hemoglobin and the efficiency of blood flow are of critical importance. Central and peripheral factors may also limit oxygen uptake, but central oxygenation depends on cardiac output and maximal arterial oxygen content.

Moreover, moving at high altitude means working in an environment with low atmospheric pressure. As a result, the oxygen content of the inhaled air is low, an atmospheric hypoxia occurs. Exercise further increases the body’s demand for oxygen and this demand must be met in the face of a drop in oxygen-driven pressure. The initial obstacle is incomplete oxygenation of the blood in the lungs. In order to ensure the delivery of oxygen to the organism, this is met by increasing the rate of blood circulation, which is a result of the constant stimulation of the sympathetic nervous system by the hypoxia at high altitude.^31^ Other consequences of sympathetic stimulation include an increase in resting metabolic rate, a shift from glycogen to free fatty acids as the primary source of energy, and increased erythropoiesis by bone marrow stimulation. The parasympathetic nervous system may also be stimulated at high altitude, which could explain the decrease in maximum heart rate.^31^

Unlike the high-altitude environment, diving operations require entering an environment with high pressure and high oxygen content, which will lead to a significant increase in symptoms caused by oxygen toxicity in the central nervous system (CNS) and lungs, and the main manifestations are headache, nausea, and coughing, and in severe cases, temporary amnesia and loss of consciousness that can threaten life.^32^,^33^ Similar situations may also occur during hyperbaric oxygen treatment (HBOT).^34^

## Oxygen in Cardiovascular Circulation

### Oxygen in the normal heart

The heart is the most active metabolic organ and has a huge energy requirement. The heart is the first organ to develop at the embryonic stage, consuming large amounts of ATP to pump oxygen-enriched blood to all other embryonic organs while tolerating a hypoxic environment. During the embryonic and neonatal periods, cardiac development goes through varying stages, including specification, morphogenesis, and maturation stages, and there are changes in cardiac physiological composition and pumping function.^35^,^36^,^37^ Oxygen is an essential component of energy metabolism in cardiomyocytes, and is necessary for the physiological and metabolic activity and development of the adult heart. As the heart transitions from a hypoxic to a normoxic environment, cardiac development matures, accompanied by a series of changes in substrate concentrations, metabolic intermediates, metabolic enzymes, and substrate transport proteins. In contrast to normal cardiac metabolism, the failing heart undergoes metabolic remodeling, a process that involves alterations in metabolic substrate utilization and oxidative stress.^35^,^38^

A study has shown that cardiac oxygen consumption is linearly related to cardiac load.^39^ The intracellular ATP concentration remains unchanged regardless of the increase in cardiac load.^40^ This extraordinary cardiac energy homeostasis may result from multiple mechanisms of cardiac energy metabolism regulation, including intracellular metabolite channeling via coupling reactions, Ca^2+^/Mg^2+^, as well as AMP signaling and metabolic microregion compartmentalization to match intracellular energy demands under conditions of OXPHOS with metabolic stability.^39^,^40^,^41^,^42^ When systemic oxygen tension is higher than normal, arterial blood oxygen has a linear negative dose-response relationship with cardiovascular function, the higher the arterial blood partial pressure of oxygen, the worse the cardiac function to some extent. This may also be a direct effect of supplemental oxygen on the vasculature, since hypoxemia can lead to increased aortic and peripheral vascular resistance.^43^ Proximal aortic and peripheral resistance is increased by hypoxemia, but reduced venous return implies additional cardiac blood pooling and compensatory relaxation of capacitance vessels.^43^

Oxygen levels play a key role in cardiac injury, as highlighted by the recent finding that hyperoxia, ROS, and concomitant DNA damage induce cell cycle exit in neonatal cardiomyocytes.^44^ During hypoxia, mitochondrial OXPHOS is inefficient and ROS are produced. To avoid the adverse effects of these compounds, myocardial hypoxia inducible factor (HIF)-1α establishes a gene expression pattern that promotes the production of large amounts of free radicals by mitochondria. The reduction of cell body biomass limits the level of OXPHOS and ROS production.^45^

### Oxygen in myocardial injury

Oxygen plays a crucial role in aerobic respiration. Cell death due to hypoxia occurs as a result of the inability of the cell to carry out aerobic respiration.^46^ In cells under hypoxic conditions, mitochondria need to consume more glucose in order to produce an equivalent amount of ATP compared to environments with a standard level of oxygen, resulting in the production of large amounts of ROS.^47^,^48^ Mitochondria are the main source of ROS in cardiomyocytes.^49^ ROS are known to disrupt mitochondrial electron transport complexes, leading to a positive feedback loop during ischemia-reperfusion or hypoxia/reoxygenation, increased ROS toxicity impairs the respiratory chain, which can lead to further massive production of ROS.^50^ Due to the hypermetabolic nature of the heart, cardiomyocytes are susceptible to hypoxic conditions, and the depletion of oxygen from damaged cardiac tissue can lead to massive death of cardiomyocytes, myocardial fibroblasts and endothelial cells in the ischemic area.^51^,^52^ During myocardial infarction, the blood supply is restricted to one part of the heart due to blockage of the coronary arteries, which in turn restricts the oxygen supply. This oxygen shortage can lead to hypoxia in cardiac tissues as well as an imbalance between the metabolic demands of cardiomyocytes and oxygen supply.^53^ Oxygen therefore plays a crucial role in cell viability and it is vital to provide the damaged tissue with an adequate supply of oxygen to prevent any further damage, protecting cardiac cells and promoting cardiac repair, which can be achieved by reintroducing oxygen to the infarcted area.^52^ The theory behind the use of hyper-oxygenation for the treatment of acute myocardial infarction is reducing the infarct area by increasing the delivery of oxygen to the damaged myocardium.^54^ This method increases the oxygen level in the blood around the damaged tissue to reverse the effects of hypoxia and avoid the damage caused by slow local circulation after myocardial infarction.^55^ Kelly et al.^55^ demonstrated that in a canine model of myocardial infarction, 100% oxygen inhalation over a short period of time reduced the infarct size by 38% and improved the cardiac function.

HBOT (inhalation of 100% oxygen at high pressures > 1 atmosphere (atm); 1 atm = 101.325 kPa) is another form of oxygen delivery. This method effectively increases the concentration of free oxygen dissolved in the plasma compared to conventional oxygen therapy,^56^,^57^ which can significantly increase cell survival in the infarcted area.^56^ Khan et al.^58^ reported in 2019 that exposing myocardial infarcted rats to hyperbaric oxygen for 90 minutes that lasted for 4 weeks, improved cardiac function after myocardial infarction and significantly reduced left ventricular end-systolic volume. On the other hand, HBOT can significantly improve conditions such as decompression sickness, chronic diabetic ulcers,^59^ transplants and flaps, delayed radiation injury, and necrotic soft tissue infections.^60^,^61^ However, there are also risks of other adverse events, including vision loss (usually temporary) and ear barotrauma.^62^ Therefore, attention should be paid to the duration of the treatment and the cycle of HBOT.

In addition, a study showed that a fraction of inspired oxygen (FiO_2_) of 40% was sufficient to reduce myocardial acid phosphokinase due to acute ischemic injury and ST-segment elevation, whereas higher FiO_2_ did not result in further improvement of the injury.^51^ However, another study demonstrated no significant differences in mortality, analgesic requirements, and infarct size between patients with suspected or confirmed myocardial infarction who received routine oxygen therapy within 24 hours of symptom onset and those who received ambient air therapy.^63^ A positive effect of oxygen therapy was also not found in several other clinical studies.^64^,^65^ In contrast to this protective effect, Wijesinghe et al.^66^ have demonstrated that in uncomplicated myocardial infarction, the routine use of high-flow oxygen instead led to a further increase in infarct size and risk of death. In 2018, a meta-analysis involving 7190 subjects showed that inhaled oxygen at normal saturation had no benefit in patients with myocardial infarction compared to the anaerobic group, and that prolonged administration of high levels of supplemental oxygen may lead to other harmful effects. For example, elevated oxygen levels in the blood also increase the formation of ROS.^65^ These excess highly reactive molecules produce oxidative stress, which ultimately leads to damage to cellular structure.^67^,^68^ Thus, based on these adverse effects of oxygen therapy and the paucity of evidence regarding the risks and benefits of this treatment, further clinical studies are necessary to assess its effectiveness.

**Figure 2:**
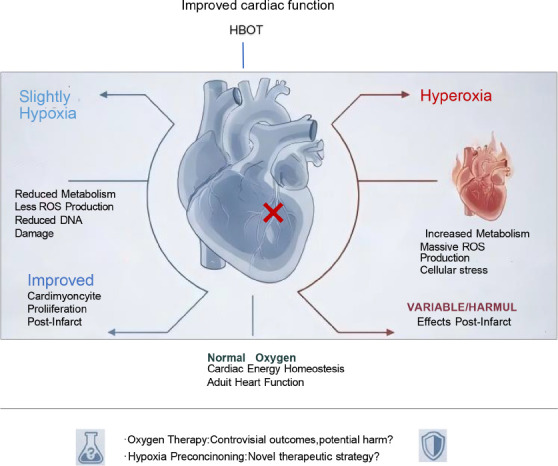
The effects of oxygen metabolism and oxygen concentration on infarcted myocardium. Due to the decreased metabolic level of the damaged myocardium, the demand for oxygen also decreases. Mild hypoxia can reduce the production of ROS and DNA damage, and even activate the regeneration of myocardial cells. High oxygen levels can lead to the generation of a large number of ROS, exacerbating the oxidative stress damage to the damaged myocardial cells. HOBT can improve the cardiac function after myocardial infarction and increase the survival rate. Routine oxygen therapy for patients with myocardial injury may have potential risks. Created with Microsoft PowerPoint. HBOT: Hyperbaric oxygen treatment; ROS: reactive oxygen species.

Interestingly, unlike hyperoxia, hypoxia (oxygen concentration below 21%) has instead been shown to have a protective effect on infarcted myocardium.^69^ Reduced mitochondrial metabolism is observed after hypoxia, with reductions in mitochondrial DNA copy number, cristae density, and protein expression of the Krebs cycle and fatty acid β-oxidases. At the same time, DNA oxidative damage in the nucleus of cardiomyocytes is also reduced. Notably, chronic hypoxia also induces cardiomyocyte proliferation after myocardial infarction, accompanied by a significant improvement in left ventricular systolic function.^70^ ROS production in mitochondria after birth inhibits cardiomyocyte proliferation.^71^,^72^ The underlying cause of heart failure after myocardial infarction is precisely the inability of the adult mammalian heart to regenerate damaged myocardium. In contrast, some vertebrate species and immature mammals are able to fully regenerate their hearts by proliferating cardiomyocytes in response to many types of myocardial injury. However, little is known about the difference between proliferating cardiomyocytes and terminally differentiated, nonproliferating cardiomyocytes. It has been shown that oxygen metabolism and oxidative stress play a key role in regulating the proliferative capacity of mammalian cardiomyocytes.^69^ Mice surviving myocardial infarction showed significant improvement in left ventricular systolic function even after recovery from hypoxia to ambient oxygen.^70^ Reducing oxygen metabolism in the adult mammalian heart may induce regeneration of adult cardiomyocytes by blunting oxidative damage, thereby improving cardiac function after myocardial infarction.^69^ The evidence showed that oxidative metabolism is a key regulator of the proliferative capacity of mammalian cardiomyocytes. Although further studies are needed to assess the safety and efficacy, hypoxia may be a novel, albeit counterintuitive, strategy for treating cardiomyopathy. From a mechanistic perspective, the molecular mechanisms underlying environmental oxygen-dependent metabolic transitions in cardiomyocytes remain poorly understood. In particular, upstream signaling pathways, transcription factors and epigenetic modifiers involved in regulating the metabolic transition from the energy-demanding non-proliferative state to the regenerative state are important future therapeutic targets (**Figure 2**).

## Oxygen in the Central Nervous System

The brain is the most oxygen-consuming organ in the human body, which relies mainly on OXPHOS for energy supply^73^ and requires a constant supply of oxygen.^74^ However, different regions of the brain require different levels of oxygen,^75^ and a steadily decreasing gradient of oxygen levels is formed when blood reaches brain tissue.^76^ Oxygen pressure plays an indispensable role in regulating various aspects of cell biology (cellular metabolism, proliferation, morphology, senescence, metastasis, and angiogenesis).^77^ In physiological states, the brain has an effective blood flow autoregulation capacity that can maintain its stable oxygen supply at different hematocrits.^78^

The specific need of neurons for oxygen makes the brain highly susceptible to neuronal apoptosis when hypoxic injury occurs, which in severe cases can lead directly to brain death. Neuroinflammation following hypoxia leads to brain damage and activation of glial cells, resulting in the production of inflammatory cytokines, such as interleukin-1β and tumor necrosis factor-α.^79^ These cytokines play an important role in both normal CNS development and in the response to brain injury. Hypoxia also induces CNS disorders by increasing oxidative stress and inflammation, affecting synaptic maturation and leading to brain damage, affecting motor coordination, sensory systems, learning and cognition, and may also lead to anxiety-like behavior, hyperactivity and sensory-motor developmental delays in animals.^80^ Under hypoxic conditions, three important barriers of the CNS: blood-brain barrier, blood-cerebrospinal fluid barrier and cerebrospinal fluid barrier, are affected to varying degrees, especially the blood-brain barrier.^81^ In humans, symptoms associated with plateau hypoxia are mainly attributed to inflammation^82^ and cerebral edema,^83^ two common sequelae of CNS barrier injury. A study has shown that hypoxia or low oxygen concentrations can strongly affect cell metabolism and fate by inducing changes in the expression levels of specific genes.^77^ Of note, in response to hypoxia, brain cells reduce their dependence on OXPHOS, upregulate glycolytic enzymes and glucose transporter proteins to give glycolysis a more prominent role in ATP production,^73^ and also activate the expression of HIF-1 proteins to minimize the deleterious effects of hypoxic events on the brain.^84^ HIF-1 is stimulated at low oxygen levels and inhibited under normoxic conditions.^84^ Numerous studies have shown that in the CNS, short periods of hypoxia have also been found to attenuate the damage caused by subsequent prolonged hypoxia.^85^ This hypoxic preconditioning leads to the up-regulation of genes related to cellular growth, signaling, metabolism, and stress-responsive pathways, and significantly improves the survival of cells in hypoxia.^86^,^87^,^88^

**Figure 3:**
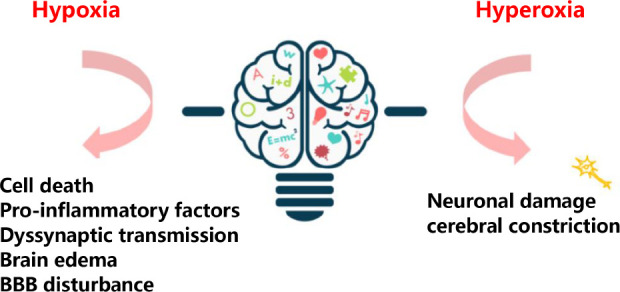
Both hypoxia and hyperoxia may cause cerebral damage. Treatment of hypoxia or hypoxia-associated diseases by supplying highly concentrated oxygen or HBOT may potentially lead to a reversal condition of oxygen overload, leading to or exacerbating neuronal damage. Created with Microsoft PowerPoint. BBB: Blood–brain barrier; HBOT: hyperbaric oxygen treatment.

When symptoms of hypoxia or hypoxia-associated diseases are present, they are usually treated by utilizing inhalation of highly concentrated oxygen or hyperbaric gas, which can potentially lead to a reversal condition of oxygen overload. That is, the supply is greater than the demand, which may lead to a number of adverse effects.^89^ A study has shown that hyperbaric or high oxygen concentrations force the production of large amounts of ROS, which can disrupt cellular physiological homeostasis.^77^ The role of ROS is to regulate a variety of signaling pathways in cellular physiology, which help to orchestrate cellular reproduction, differentiation, migration and angiogenesis.^90^,^91^ Whereas excessive ROS can lead to oxidative stress, which is a major contributor to various deleterious mechanisms that promote apoptosis in CNS derivatives,^92^ and result in damage to cell membranes, alter protein structure and function, lipid denaturation and DNA alterations, lipid degeneration, and structural damage to DNA, among other hazards.^93^ The brain, as a major organ of oxygen metabolism, is particularly susceptible to these deleterious effects.^94^ Short periods of hyperbaria or hyperoxia induce vasoconstriction, which reduces cerebral blood flow, thus exacerbating hypoxia in the central system.^95^ Clinical studies have found that high oxygen levels cause neuronal and glial cell death, leading to the white and grey matter damage observed in preterm infants. At critical stages of brain maturation, hyperoxia alters developmental processes, leading to disruption of neuroplasticity and myelin formation.^96^ In preterm infants, prolonged or fluctuating exposure to supraphysiological levels of oxygen may lead to preterm encephalopathy with cystic or diffuse periventricular leukoencephalomalacia, compared to intrauterine disorders.^97^,^98^ Whereas in terms of infants, birth asphyxia followed by hyperoxia resuscitation may also significantly increase mortality and morbidity.^99^ In conclusion, the advantages and disadvantages of administering hyperoxia immediately after brain hypoxia need to be fully taking into consideration (**Figure 3**).

## Impacts of Age and Sex on Oxygen Demand and Usage

It is well known that most oxygen is consumed in the mitochondria. However, several studies on rodents have shown that mitochondrial oxygen consumption and the efficiency of OXPHOS decrease with age.^100^,^101^ This has also been observed in humans. Older subjects showed significantly higher lactate levels at all exercise time points, which was also attributed to the decrease in mitochondrial oxidative metabolism with age.^102^,^103^,^104^ Interestingly, some animal studies have shown that in subjects with impaired mitochondrial function, appropriately low concentrations of oxygen (lower than that in air, e.g., 15%) are more beneficial to longevity than high or even normal concentrations (21%) of inspired oxygen.^105^,^106^,^107^ When aging is coupled with impaired mitochondrial metabolism, even normal oxygen (21%) may become a state of relative oxygen excess, as inhaled oxygen could not be fully metabolized due to decreased mitochondrial oxygen consumption capability. Hyperoxia or relative hyperoxia can cause deleterious hemodynamic effects, including peripheral and coronary vasoconstriction and direct cytotoxicity through the production of ROS.^108^ There is ample evidence that mitochondria play an important role in the aging process, and the function of the ETC, located in the inner membrane of the mitochondria is a major thrust in the study of aging. It is not only a source of energy for the metabolic processes of the cell, but also a major generator of ROS.^109^,^110^ Studies have demonstrated the partial pressure of arterial oxygen (PaO_2_) in the elderly is lower than that in the young, and there are gender differences.^111^,^112^ For instance, amongst older people aged 70 years and above, the mean normal PaO_2_ value for men is 77 ± 9.1 mmHg (lower limit of normal is 62 mmHg), whereas in women the mean PaO_2_ value is 73.5 ± 8.4 mmHg (lower limit of normal is 59.6 mmHg).^111^ Since the normal reference range of PaO_2_ decreases with age,^112^,^113^ in the oxygen therapy for the elderly population, the oxygen concentration should be more precisely targeted and controlled.

Furthermore, aging significantly reduces mitochondrial oxygen consumption in both male and female hearts. However, mitochondrial oxygen consumption is significantly increased in both mature and aged female hearts compared to male hearts.^114^ Hyperoxia can also negatively affect the elderly, but there is significant variability in the stress response to hyperoxia in males and females.^115^ Interestingly, compared to young adults, pre-pubertal healthy male and female children have no sex difference in mean blood oxygen saturation.^116^ It is suggestive that this difference is most likely due to age-related changes in sex hormone levels. Further research is needed to explore the mechanisms that may explain why preadolescent children do not show sex differences in oxygen saturation compared to adults. The damage caused by high oxygen also varies by sex. A study has shown that female mice are more susceptible to hyperoxygen-induced lung damage than males, alongside severe bradycardia and high mortality rates in the females.^117^ Furthermore, under hyperoxic conditions, females have a shorter mean survival time than males.^118^ A clinical report has shown that females are more likely to develop acute respiratory distress syndrome than males,^119^ and have a significantly higher mortality rate than males in mechanically ventilated patients under hyperoxia (90% oxygen).^117^ Conversely, hyperoxia-induced lung injury appears to be more severe in males,^120^ and the mortality rate among premature infants diagnosed with bronchopulmonary dysplasia is also higher in males than in females.^120^ In neonates, hypoxia is tolerated to some extent, but resistance against hyperoxia is poor.^121^ In preterm and immature infants, even a few minutes of exposure to pure oxygen in the delivery room may have deleterious effects.^122^ This is likely because excess oxygen may cause oxidative stress and damage to vital organs. Preterm infants, especially immature newborns, are more susceptible to oxidative stress than term infants because they have poorer defenses against oxidative stress. A study of the effects of hyperoxia exposure on cardiac pathophysiology in male and female mice shows that hyperoxia can produce cardiotoxicity, resulting in increased serum myocardial markers such as cardiac troponin I and lactate dehydrogenase.^117^

## Oxygen Supply in Critically Ill Patients

Due to the fact that hypoxemia is directly associated with increased mortality and the occurrence of serious adverse events,^123^,^124^ medical oxygen has been widely used for patients, especially those under intensive care. However, its advantages and disadvantages remain controversial under certain conditions. Oxygen therapy is usually considered useful to treat or prevent hypoxia. However, the efficacy of oxygen therapy is largely dependent on the physiological characteristics of hypoxia.^5^ Oxygen therapy can reverse tissue hypoxia, which in many cases can be life-saving, but its overuse may lead to hyperoxemia, where PaO_2_ is higher than normal physiological levels.^125^,^126^

Currently, high-flow nasal cannula oxygen therapy (HFNC) is recommended by guidelines for the treatment of acute hypoxic respiratory failure in the intensive care unit (ICU),^127^,^128^ and its use as a flow-dependent upper airway carbon dioxide removal modality helps to reduce anatomical dead space ventilation and carbon dioxide rebreathing.^129^ HFNC brings actual FiO_2_ closer to the set FiO_2_ than low-flow oxygen therapy.^130^ In addition, HFNC significantly reduces nasopharyngeal resistance, allowing gases to pass more smoothly into the lower airway. When the patient has a low tidal volume and a low ventilation rate, a high flow rate of 60 L/min keeps the small airways open by maintaining a low level of positive airway pressure, which allows the exchange of oxygen and CO_2_.^131^ However, it was found that there was no significant difference in the incidence of hypoxia between oxygen flows at 30 and 60 L/min during HFNC when being used at the same stable FiO_2_.^132^ Moreover, HFNC may have a short-term effect of promoting respiratory muscle recovery through inhalation gas conditioning, humidification and heating of the inhaled air, which can promote ciliated mucociliary clearance and reduce chronic airway inflammation, among other protective effects.^133^,^134^ However, among children aged 1–4 years with acute hypoxic respiratory failure, the ICU admission rate in the HFNC group was significantly higher than that in the standard oxygen inhalation group (12.6% *vs*. 6.9%), and the total hospital stay was also longer.^135^ Furthermore, in patients with chronic obstructive pulmonary disease, if their resting saturation of peripheral oxygen (SpO_2_) is greater than 88%, long-term oxygen supplementation treatment does not prolong the survival time of the patients compared to no long-term oxygen supplementation treatment, however, when the resting SpO_2_ is 88% or lower, long-term oxygen supplementation treatment may be more harmful.^136^

In addition, in patients with coronavirus disease 2019, both conventional oxygen therapy and non-invasive positive pressure ventilation improved oxygenation and reduced the likelihood of intubation.^137^,^138^ However, hyperoxia-mediated oxidative lung injury may further exacerbate oxygenation deficits and may paradoxically drive the need for higher FiO_2_.^139^ There is evidence that free oxygen therapy increases mortality without improving other outcomes and that supplemental oxygen may become more detrimental in the SpO_2_ range of 94–96% and above.^113^,^140^ A study involving ICU patients suggested that mechanically ventilated patients should be closely monitored for PaO_2_ and FiO_2_, especially during the early to mid-term period after ICU admission, and prolonged (more than 28 days) inhalation of higher concentrations of oxygen is associated with an increased risk of death.^125^ However, another study involving ICU patients with mechanical ventilation showed that compared with the conventional oxygen therapy group, the conservative oxygen therapy group (SpO_2_ target of 90%, range 88–92%) did not significantly reduce the all-cause mortality within 90 days.^141^ The finding of no significant difference between the two groups’ results might be due to the varying oxygen requirements of different diseases. For instance, using a lower SpO_2_ target can reduce the mortality rate of patients with acute brain injury, while using a higher SpO_2_ target can reduce the mortality rate of patients with sepsis and abnormal vital signs, and implementing individualized oxygenation targets may lower the total mortality rate of critically ill patients on mechanical ventilation.^142^

The effect of high FiO_2_ on mortality is partly mediated by hyperoxemia, while high FiO_2_ can also have a direct effect on mortality. Additionally, it is worth noting that inhaling high concentrations of oxygen may also cause partial or complete denitrogenation of the alveoli, resulting in alveolar collapse and atelectasis.^143^ This phenomenon is particularly common during general anesthesia with endotracheal intubation.^144^,^145^ A study on patients with acute trauma showed that the incidence of atelectasis in the restrictive oxygen supply group (arterial oxygen saturation target of 94%) decreased significantly compared with the free oxygen supply group.^146^ Clinical guidelines also recommend that most acutely hospitalized patients should be maintained with an SpO_2_ no higher than 96%.^147^ Hypoxemia has life-threatening effects in a dose-dependent manner.^125^,^148^ Oxidative stress induced by hyperoxia exposure is time- and dose-dependent^43^ and both the duration of exposure to hyperoxia and the dose size are related to the severity of the harm.^125^

## Oxygen Toxicity

Oxygen has specific biochemical and physiological effects, and although there is an adequate range of effective/safe doses, either too low or too high a concentration of oxygen can be extremely harmful. Acute hypoxia increases heart rate by stimulating sympathetic nerves and reduces systemic vascular resistance by releasing vasodilatory metabolites through tissues and endothelium.^149^ In contrast to the peripheral circulation, hypoxia also induces pulmonary vasoconstriction,^150^ leading to an increase in right ventricular afterload, which in turn leads to right ventricular failure.^151^ In addition, exposure to chronic hypoxia has been associated with adverse pulmonary vascular remodeling and the development of fixed precapillary pulmonary hypertension.^108^ Furthermore, hypoxia can have deleterious effects on acute, chronic and intermittent time scales. Acute, severe hypoxic exposure may occur when healthy individuals rapidly ascend to high altitudes (2500 m or higher) and can cause acute altitude sickness, high altitude cerebral edema and high-altitude pulmonary edema.^152^ In recent years, Yin et al.^153^ developed a stable rat model of high-altitude pulmonary edema using the transparent chamber, which may be more conducive to further explore the mechanism of high-altitude pulmonary edema.

The oxygen toxicity of hyperoxia was demonstrated in animal studies as early as in the late 1800s, demonstrating the oxygen toxicity of hyperbaric hyperoxia to the CNS, and Smith^154^ further described the pulmonary toxicity of normal oxygen (above 21%, 1 atm), acute lung injury due to hyperoxia. Pathological features of hyperoxia acute lung injury include damage to the pulmonary capillary endothelium, death of alveolar type I epithelial cells, hypertrophy of type II epithelial cells, interstitial edema, accumulation of neutrophils, decreased production of alveolar surface-active substances, and decreased lung compliance.^155^ A study has shown that small animals such as mice can tolerate moderate to high oxygen concentration levels (40–80%) for more than a week, but still higher levels (80–100%) of oxygen is fatal to the animals within a few days.^154^ In humans, exposure to hyperoxia conditions is routinely used as oxygen therapy to address problems such as blood hypoxemia and tissue hypoxia in a variety of pathological conditions, but the harmful effects of oxygen toxicity still need to be approached with caution. Oxygen therapy has been widely used in recent years for the treatment of patients with severe coronavirus disease 2019, but these patients have severe lung damage later in life and impair functions such as gas exchange.^156^ In neonates, hyperoxia also interferes with lung development, leading to developmental abnormalities that persist into adulthood. Animal models, particularly mouse models, have been used extensively to study the effects of hyperoxia in neonatal lungs.^157^ In addition, premature infants lack sufficient antioxidant capacity and are more susceptible to oxygen toxicity, and this acute injury leads to long-term pathologies such as bronchopulmonary dysplasia. Many neonates treated with oxygen develop other serious complications such as blindness and abnormal brain and lung development due to chronic hyperoxia exposure.^158^

Hyperoxia contributes to cell death through a number of pathways and can promote excessive production of ROS.^158^ Oxidative DNA damage can occur directly as a result of base modifications or strand breaks, and both nuclear and mitochondrial DNA can be affected by hyperoxia. Proteins are also important targets of oxidation, which has important effects in both physiological signaling and pathological processes.^158^ Hyperoxia impairs cellular function by causing oxidative damage to macromolecules and dysregulation of cellular signaling processes. Under hyperoxic conditions, ROS alter multiple signaling pathways such as Nrf2,^159^ nuclear factor κB and mitogen-activated protein kinase pathways.^160^,^161^ Oxidative DNA damage driven by hyperoxia also triggers the activation and upregulation of p53, leading to cell cycle arrest, reduced proliferation, senescence or cell death.^162^,^163^ Inhibition of OXPHOS under hyperoxia conditions is also associated with the inhibition of several key enzymes. For example, the activity of the pyruvate dehydrogenase complex is reduced in the lungs of mice and rats exposed to 95–100% oxygen.^164^ Inactivation of the pyruvate dehydrogenase complex limits pyruvate oxidation in the TCA cycle. In contrast, α-ketoglutarate dehydrogenase) can be similarly inhibited by hyperoxia. In addition, hyperoxia inhibits the activity of mitochondrial respiratory complexes I and II.^165^ Thus, almost all organs and tissues may be targets of oxygen toxicity (**Table 1**).

**Table 1:** The main characteristics of oxygen toxicity

Category	Main mechanisms and consequences
**Systemic impact**	
Cell membrane damage	ROS cause lipid peroxidation, which damages the integrity and function of cell membranes
Loss of protein function	Oxidizing key groups leads to enzyme inactivation and structural protein denaturation, affecting cell function
DNA damage	Attacking DNA molecules may lead to gene mutations, apoptosis or cancer
Mitochondrial dysfunction	Disrupting the energy metabolism center leads to the depletion of cellular energy (ATP) production
Systemic inflammatory reaction	Damaged cells release inflammatory factors, triggering a chain of inflammation and exacerbating tissue damage
Exhausted antioxidant system	Long-term high-oxygen exposure depletes the body's antioxidants, causing a decline in the body's defense capabilities
**Central nervous system toxicity**	
Convulsions and epileptiform seizures	Inhibiting glutamic acid decarboxylase leads to a reduction in GABA synthesis and an increase in glutamic acid
Vision disorder	The visual center and optic nerve are affected, resulting in blurred vision.
Auditory abnormality	Damage to the auditory center leads to tinnitus and hyperacusis.
Muscle twitching and dizziness	May result in strong constriction of blood vessels in the brain, reducing the blood supply to the inner ear's vestibular system.
**Pulmonary toxicity**	
Destruction of the alveolar capillary barrier	Damage alveolar and capillary cells, leading to pulmonary edema
Decreased surface active substance	Damage to type II cells in the alveoli leads to alveolar collapse and a decrease in lung compliance.
Pulmonary fibrosis	The direct attack by ROS and the infiltration of inflammatory cells prompt the damaged alveolar epithelial cells to secrete pro-fibrotic factors (TGF-β1), thereby driving pulmonary fibrosis.
Induce or aggravate ARDS	Pulmonary edema and alveolar collapse
Pulmonary atelectasis	After high-oxygen mediated nitrogen "flushing out" nitrogen, absorption of the oxygen in the alveoli causes the alveoli to collapse
**Ocular toxicity**	
Retinopathy of prematurity	Abnormal proliferation and contraction of retinal blood vessels in premature infants are the main cause of blindness
Adult myopia or posterior fibrous hyperplasia of the lens	Long-term hyperbaric oxygen may cause refractive changes in the lens and fibrous tissue hyperplasia
**Other special risks**	
Cardiovascular toxicity	This leads to peripheral blood vessel constriction and reduced coronary blood flow, posing a high risk for patients with heart disease.

ARDS: Acute respiratory distress syndrome; ATP: adenosine triphosphate; GABA: gamma-aminobutyric acid; ROS: reactive oxygen species; TGF-β1: transforming growth factor β1.

## Conclusion and Outlook

There is no doubt that oxygen therapy is important and has saved many lives. However, over- or under-dosing of oxygen should be avoided. Inadequate oxygenation can have harmful effects and even endanger lives. Continuous oxygen therapy when it is no longer needed can prolong hospital stays and increase the cost of care. When assessing the effectiveness of oxygen therapy, it is important to ensure that oxygen levels and cardiac output are adequate.

Elevated levels of ROS produced by cells during over-oxygenation therapy can cause oxidative damage, and every effort should be made to correct hyperoxia and tissue hypoxia while avoiding or reducing oxygen toxicity. Research on reducing hyperoxia injury has addressed key aspects including ROS production/neutralization, apoptotic cell death and inflammatory responses. The body has multiple oxygen-sensing mechanisms to protect against hypoxia and hyperoxia to ensure an appropriate balance between oxygen supply and demand and to prevent organs and cells from suffering from hyperoxia-induced oxidative stress. A regulating system that is based on oxygen homeostatic mechanisms and cellular oxygen sensing systems should help to ensure an appropriate balance between oxygen supply and demand. Relatively conservative oxygen therapy with careful monitoring seems to be necessary and may improve outcomes and avoid harmful oxidative stress damage caused by excessive oxygen administration.^5^ Therefore, in order to prevent oxygen toxicity, we have provided some suggestions for prevention and oxygen supply (**Table 2**).

**Table 2:** The main measures for preventing oxygen toxicity

No.	Main preventive measures
1	Strictly control the oxygen concentration, oxygen partial pressure and exposure time; when using HBOT, pay attention to the its duration and cycle.
2	Professional medical monitoring; monitor SpO_2_ as a surrogate for arterial oxygen saturation
3	Use reliable equipment for monitoring and conduct regular calibration of oxygen concentration and oxygen flow.
4	Individualized oxygen supply plan: (a) initiate oxygen only when it is below the SpO_2_ lower limit; (b) titration of oxygen delivery to maintain SpO_2_ within the target range; (c) cessation of oxygen delivery when the upper limit of SpO_2_ is exceeded in order to prevent hyperoxia;
5	Carry out intermittent oxygen inhalation
6	Comprehensive health assessment and screening
7	Use a verified oxygen inhalation scheme
8	Understand and quickly identify the symptoms of early oxygen poisoning
9	Establish clear emergency handling procedures

HBOT: Hyperbaric oxygen treatment; SpO_2_: saturation of peripheral oxygen.

It should be noted that there are some limitations in our review. Firstly, the oxygen toxicity knowledge summarized in our review may not be comprehensive. Secondly, the oxygen supply plans we provided may be helpful for healthy adult individuals, but further prospective studies are needed to confirm their effectiveness in different populations. In conclusion, preventing oxygen toxicity is just as crucial as preventing hypoxia. During the process of oxygen delivery, continuous real-time personalized monitoring and guidance are particularly important, especially in the elderly.

## Data Availability

*Not applicable.*
